# Wild crickets can adjust escaping speed under varying predation cues

**DOI:** 10.1093/beheco/arag029

**Published:** 2026-03-11

**Authors:** Ruonan Li, Emily Gilford, Rolando Rodríguez-Muñoz, Tom Tregenza

**Affiliations:** Centre for Ecology and Conservation, University of Exeter, Penryn, Cornwall, TR10 9FE, United Kingdom; Centre for Ecology and Conservation, University of Exeter, Penryn, Cornwall, TR10 9FE, United Kingdom; Centre for Ecology and Conservation, University of Exeter, Penryn, Cornwall, TR10 9FE, United Kingdom; Centre for Ecology and Conservation, University of Exeter, Penryn, Cornwall, TR10 9FE, United Kingdom

**Keywords:** antipredation, tradeoffs, escape speed, developmental differences, predation cue intensity, behavioral plasticity

## Abstract

Predator-prey dynamics impose strong selection pressures on the evolution of antipredator strategies, which constitute a fundamental adaptive response in wild animals. However, these strategies incur energetic costs, requiring tradeoffs with other fitness-related traits. While predator-prey interactions have been extensively studied, empirical evidence for behavioral plasticity in responses across predation risk gradients remains limited. We conducted field experiments on wild cricket (*Gryllus campestris*) nymphs in northern Spain, to investigate (1) how the intensity of predation cues influences escape speed adjustments and (2) whether antipredator strategies vary by sex, developmental stage and according to thermal conditions. We simulated 2 levels of vibrational cues: low-intensity and high-intensity. Contrary to our threat-sensitivity predictions, nymphs exhibited slower escape responses to high-intensity cues. This pattern may reflect ecological relevance: cricket predators are primarily small vertebrates which will generate lower intensity vibrational cues than passing ungulates. Consistent with our previous findings in adults, escape speed in nymphs increased with body temperature. However, sex-dependent variation was observed only in adults, not in nymphs, suggesting developmental differences in antipredator strategies.

## Teaser

Wild animals must balance rapid escape from predators with the costs of unnecessary responses. We tested how wild field crickets adjust their escape speed in response to predator-like vibrations of different intensities and under varying temperatures. Crickets moved more slowly in response to stronger vibrational cues, while escape speed increased with body temperature. These results show that escape behavior in the wild is flexible and influenced by environmental conditions.

## Introduction

Antipredator behavior is a critical adaptive strategy for wild animals. Extensive research has explored predator-prey systems, demonstrating a strong relationship between antipredator responses, survival rates, and overall fitness (Stoks et al. 2003; Losos et al. 2004; Pérez-Tris et al. 2004; Lind and Cresswell 2005). Animals with more effective antipredator behaviors generally have higher survival. For instance, studies on wood pigeon (*Columba palumbus*) and Thomson's gazelle (*Gazella thomsoni*) demonstrate that rapid response to attack increases survival probability (Kenward 1978; FitzGibbon 1989); Juvenile redshanks (*Tringa totanus*) foraging in an exposed salt marsh environment experience a very low probability of capture per attack by a sparrowhawk (*Accipiter nisus*) due to the effectiveness of their antipredation behaviors, such as avoidance and flocking (Cresswell 1994); and small brown planthoppers (*Laodelphax striatellus*) showed elevated escape success owing to more rapid responses following prior exposure to predation risk (Wen and Ueno 2021). However, antipredator responses inevitably incur costs in terms of time or energy, which must compete with allocation to other fitness-related traits, such as foraging, somatic maintenance, resistance to pathogens and reproduction.

Animals at risk of predation (“prey”) can respond to a range of different types of predator cues (eg, visual cues, vocalizations, chemical cues, etc) (Kats and Dill 1998; Brown et al. 2004). There is clear evidence that prey adapt their responses according to the types of predator cue that they receive (see review by Jones et al. 2024). For instance, Blumstein et al. (2000) employed predator models and auditory playback of recorded predator sounds and observed that tammar wallabies (*Macropus eugenii*) exhibited a response to the visual but not auditory cues of the presence of predators. Dangles et al. (2006) used a puff of air to simulate wolf spider attacks on wood crickets and demonstrated that the prey's probability of escape can be predicted by factors such as the distance between predator and prey, the predator's approach velocity, and the signal strength. However, we have much less insight into the extent to which wild animals in their natural environment may adjust not only to the type of predation cue received, but also to the intensity of that cue.

Studies across taxa have demonstrated the occurrence of predator-induced morphological defences, which could be interpreted as evidence of modulating predator responses in relation to the stimulus. For instance, *Daphnia longispina* have been shown to develop neck teeth in response to kairomone exposure from the predator, *Chaoborus sp.* (Sperfeld et al. 2020). This phenomenon has been observed in multiple *Daphnia* species (Juračka et al. 2011; Riessen and Gilbert 2019). Similarly, studies on freshwater fish have revealed that the presence of pike (*Esox lucius*), a predatory fish, induces morphological changes in both perch (*Perca fluviatilis*) and roach (*Rutilus rutilus*). Specifically, perch exhibit increased body depth as a response to pike cues, which is associated with reduced activity, whereas roach develop a deeper caudal peduncle, a response linked to increased activity (Christensen and Persson 1993; Eklöv and Jonsson 2007). Additionally, a study on larvae of the water frog (*Rana ridibunda*) demonstrated that exposure to predatory dragonfly larvae (*Aeshna sp.*) leads to the development of larger tails and shorter but more muscular legs (Buskirk and Saxer 2001). These studies collectively highlight that prey organisms can modify investment in morphological defences in response to predation risk.

Although some studies on aquatic animals have shown that antipredator responses intensify with increased predator chemical cue concentrations (Dupuch et al. 2004; Zhao and Chivers 2005), research on how terrestrial animals, particularly insects, respond to varying predator cue intensities remains limited. Understanding how prey allocates time and energy in response to varying predator cue intensities is essential for elucidating the ecological significance of predator-prey interactions. It also has clear significance for understanding how anthropogenic disturbances may alter the noise environment and thereby affect animal populations. If prey responses tend to increase in proportion to the strength of the cue they receive, then it may be straightforward to predict the relative impact of disturbances of different magnitude. Alternatively, because anthropogenic noise can either mask or enhance signal transmission depend on habitat structure and disturbance type, the relationship between responses and the strength of the cue maybe more complex.

Escape behavior is a common antipredator strategy observed across taxa (Dangles et al. 2006; Turesson et al. 2009; Binazzi et al. 2011; Lagos 2017). As a key indicator of escape performance, higher escape speed will typically require greater muscle power and energy investment. Because resources in natural environments are often limited, animals need to allocate their energy efficiently to optimize their fitness. Reducing energy expenditure on escape behaviors in low predation risk scenarios may allow individuals to allocate more resources toward other fitness-related traits. Tradeoffs between antipredation investment and other traits have been identified in a number of species. For instance, a study on common lizards (*Lacerta vivipara*) revealed a tradeoff between the risk of parasite infection when lizards are less active and the risk of predation when they are more active (Clobert et al. 2000), which suggested an energy allocation tradeoff between antipredation strategies and immune function. Likewise, Adamo (2022) illustrates how limited resources force animals to balance antipredator defences with other physiological demands, such as immunity and detoxification. The study on Atlantic silverside fish (*Menidia menidia*) demonstrated that maximizing energy intake and growth rate incurs fitness costs, such as increased vulnerability to predation (Lankford et al. 2001). A modeling study using the evolutionary ecosystem simulation (EcoSim), showed that high predation risk suppressed prey foraging activity, increased overall movement, and decreased reproduction compared with low-risk scenarios (Khater et al. 2016), However, there remains a significant gap in understanding how prey mediate tradeoffs in behavioral responses to variation in predator cues.

To investigate whether a model wild insect adjusts its allocation of effort in behaviorally response to predator cue intensity, we conducted a field-based experimental study on nymph field crickets (*Gryllus campestris*) in a meadow in N. Spain. Field crickets are known to detect and react to both substrate-borne vibrations and air movement (Kiuchi et al. 2023). In our long-term field observations, we have recorded hundreds of attacks on crickets from a range of predators, including robins, shrews, lizards, and a range of invertebrates (Rodríguez-Muñoz et al. 2011). Escape responses were almost always immediate, involving rapid retreat into their burrows. From our own experience, an observer sitting on the ground directly adjacent to a burrow can wave their hand 30 cm above a cricket standing outside its burrow and it will not show any signs of disturbance providing there is minimal air movement. However, any shift in the observer's position that is transmitted through the substrate elicits an immediate escape response down the burrow by the cricket. There is no other plausible explanation for this very rapid running down the burrow, other than that it is an antipredator response. Previous research also indicates that crickets are highly sensitive to air currents or substrate vibrations, responding with rapidly escaping behavior or running down into a burrow (Dambach 1989; Stevenson et al. 2000; Dangles et al. 2006; Broder et al. 2021; Li et al. 2025). We therefore simulated a vibrational potential predator cue at 2 different intensity levels, strong and weak. Higher rates of acceleration require greater energy input, and weaker cues might be associated with a more distant or less deadly predator. Hence, our prediction was that individuals exposed to the strong predator cue will run faster than those exposed to the weak predator cue.

In a previous study of antipredator response in adult crickets (*G. campestris*), we found that escape speed was higher at elevated body temperatures and that there was an interaction between this relationship and the sex of the adult fleeing from a stimulus: females exhibited increased escape speeds at higher body temperatures, but males did not (Li et al. 2025). Sexual selection pressures and reproductive investment priorities are expected to differ drastically between adult males and females (Promislow 2003; Bonduriansky et al. 2008; Houslay et al. 2015). Hence these sex-specific effects of body temperature on flee speed may be driven by reproductive tradeoffs. In contrast, nymphs of both sexes are expected to prioritize survival and developmental growth, so we might not expect any sex-specific effects in nymphs. A secondary aim of our study is to investigate whether the previously observed sex-temperature interaction in escape speed in adults is already present in nymphs or develops later, potentially shedding light on the ontogeny of escape strategies in response to predation risk.

## Methods

### Study system

Our study site is the “WildCrickets” meadow in Gijon, northern Spain where we have been studying a wild population of the field cricket *Gryllus campestris* for many years (for further information, see www.wildcrickets.org) (Tregenza et al. 2022). *G. campestris* has annual generations and exhibits thermoregulatory behavior (eg, basking outside their burrows in sunny weather) (Alexander 1968; Rodríguez-Muñoz et al. 2023; Gardner et al. 2024a, 2024b). Nymphs regularly emerge from burrows to forage and bask during daylight periods and typically stay within a few centimeters of the burrow entrance. Both sexes excavate burrows from an early age, entering diapause over the winter. They then reemerge in the following spring and continue to grow, becoming more active after undergoing emergence to adulthood in April and May (Rodríguez-Muñoz et al. 2011). Both sexes are flightless and spend the vast majority of their lives within a few cm of a burrow which provides a refuge from inclement weather and from predators that are unable to squeeze into the narrow tunnel.

In March 2024, we searched the meadow for cricket burrows and identified each 1 with a flag carrying a unique number. We then installed infrared digital video cameras above 140 of the burrows, monitoring any activity 24 h a day and allowing us to track the behavior and developmental stage of crickets.

### Escape speed measurement and predation cues stimulus

We conducted measurements following the methodology described in Li et al. (2025). Briefly, a 240 frames per second, 2.7 K pixel GoPro was mounted on a tripod so that it was 50 cm above the substrate and facing directly downwards above the burrow. In the video recordings, the length of the cricket occupied ∼5% of the height of the frame. For each trial, the operator provided an artificial vibrational stimulus by dropping a ball down a 50 cm long plastic tube with an internal diameter of 43 mm. The bottom end of the tube was kept 15 cm from the substrate, ∼10 cm from the burrow, always positioned behind the direction of the burrow opening. To facilitate movement distance measurements from the video, we placed a ruler on the ground adjacent to the burrow during the experimental recordings which served as a reference for distance measurement. We conducted trials on each burrow using 2 different kinds of balls, a 40 mm diameter, 9 g cork ball, to provide the weak stimulus and a 40 mm diameter, 266 g steel ball to provide the strong stimulus.

The stimulus consisted of several components, including the possibility of subtle vibrations generated as the ball was released through the plastic tube and transmitted to the substrate via the legs of the tripod, an air current produced while the ball traveled down the tube before striking the ground, the impact upon contact with the substrate, and a brief rolling phase that followed. Although each of these components may contribute to triggering the crickets’ escape behavior, our observations indicate that the vast majority of responses occurred immediately after the initial impact of the ball on the substrate, suggesting that this “thud” serves as the primary trigger for the escape behavior in most trials. Natural predators are expected to generate more complex and temporally patterned vibrations; our artificial cue is not intended to represent the complete structure of a specific predation cue in the wild. Our aim was to produce a generic threat cue that likely overlaps substantially with natural predator cues, given that wild crickets typically detect predation risk through substrate vibrations.

The majority of our data (*n* = 137) were collected by consecutively dropping both types of balls in the same day on each cricket, with the order of ball type alternated between trials to minimize potential order effects. Specifically, if a cork ball was dropped first followed by a steel ball on the first cricket, the order was reversed for the second cricket, and then switched back for the third cricket, and so on. A smaller subset of the data (*n* = 72) was collected by first dropping one type of ball on a given cricket, allowing it to rest for 1 d, and then dropping the second type of ball the next day.

The operator set up the camera and vibration stimulus apparatus during daylight hours between 09:00 and 18:00 and sat near the burrow as motionless as possible waiting for the cricket to come out of burrow. Once a cricket emerged from its burrow and assumed a stable position (not moving for ∼1 min), the operator measured its body temperature using an IR thermometer (either RS PRO DT-836 IR thermometer, see https://www.rs-online.com/, or Testo 830-T4 IR thermometer, see https://www.testo.com/). They then started the video camera recording using a voice command trigger and immediately afterwards released the stimulus ball.

We used the software “Kinovea” (https://www.kinovea.org/download.html) to review videos in slow motion using the frame number displayed with each frame representing 1/240th of a second. In each video using the ruler placed beside the burrow and the line annotation tool in the software, we marked out the initial 1.5 cm distance to avoid the influence of expected accelerate as they run. This escape distance has been previously validated as a reliable measure for assessing escape speed in our earlier study (Li et al. 2025). We then extracted the parameters below: (1) the frame in which we detected the first obvious movement of the target cricket (F_1_); (2) the frame where the cricket reached a distance of 1.5 cm from its original position at the furthest distance away from the burrow (F_2_); (3) the initial escape orientation of the crickets relative to their burrow (head toward vs. tail toward the burrow). We then calculated escape speed (in m/s) using the formula:


$$V=0.015/(F_{2}\text{–}F_{1})\times (1/240)$$

where F1 and F2 represent the frame numbers at the start and end of the escape movement of 1.5 cm distance, respectively, and 240 fps is the frame rate of the video recording.

### Data analysis

The dataset comprises 89 cricket nymphs (*F* = 44, *M* = 45) with a total of 209 measurements (*F* = 102, *M* = 107), averaging 2.35 measurements per individual (SD = 1.26). Three measurements where the sex of the cricket could not be determined were excluded from the dataset. Individual ID was included as a random effect in all analyses to avoid pseudo-replication.

We used the “lme4” package (1.1.36) in R (R Development Core Team, 2024, v. 4.4.2) and RStudio (RStudio Team, 2023, v. 2023.03.0) to build linear mixed models (LMMs). Escape speed was the response variable, with body mass, orientation, body temperature, sex and stimulus categories and the interactions of body temperature with sex and stimulus as predictors, and ID as random effect in the model. To aid model convergence and facilitate the interpretation of standardized effect sizes, body mass and body temperature were *z*-transformed using the scale() function in R. Our escape speed data fitted a normal distribution checked via histograms and QQ-Plots, the model residuals were checked for normality and absence of multicollinearity.

## Results

### Do crickets exhibit differential responses to varying predation cue intensities?

**Figure 1:**
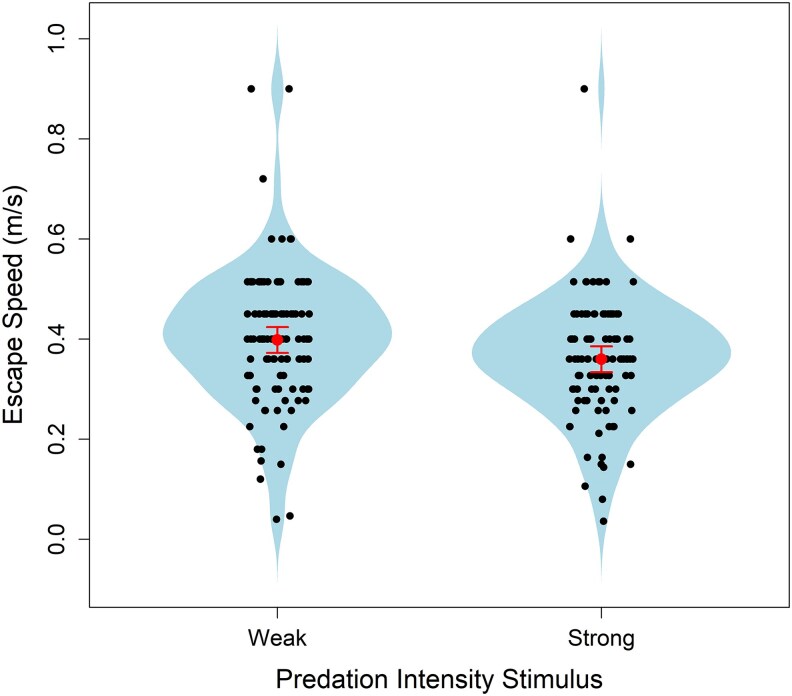
Bean-plot of the relationship between predation intensity stimulus (dropping a ball) and escape speed in wild field cricket (*G. campestris*) nymphs. Weak represents a cork ball, while strong represents a steel ball. Least-squares means are indicated by points with error bars representing 95% confidence intervals.

**Figure 2:**
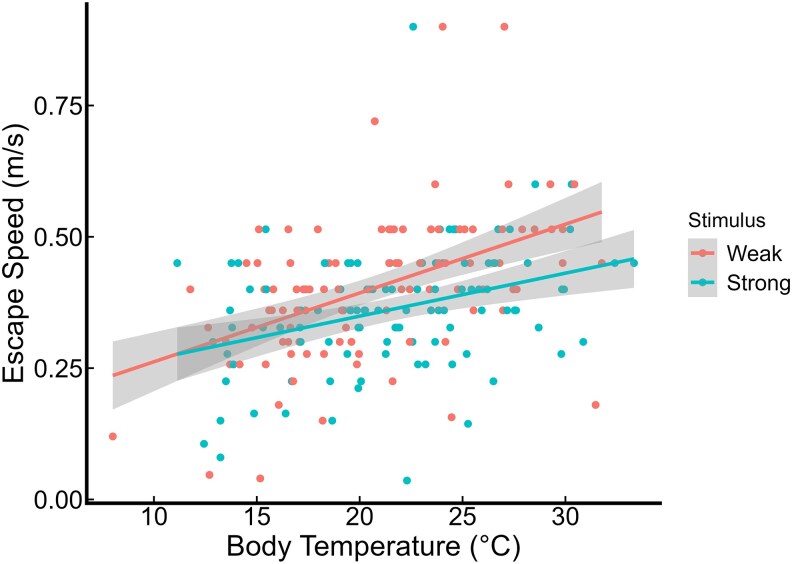
The relationship between body temperature and escape speed under 2 predation cue intensities. Red points and lines correspond to weak stimulus, and blue points and lines to strong stimulus. Shaded areas indicate 95% confidence intervals.

We found that crickets in the strong stimulus group ran significantly slower compared with those in the weak stimulus group, and that greater body mass was associated with increased escape speed (Table 1, Fig. 1). Additionally, we observed a positive relationship between body temperature and escape speed (Fig. 2), which shows that crickets run faster when their body temperature is higher.

**Table 1:** Results of a linear mixed-effects model for the influence of body temperature, stimulus type, sex, body mass, orientation and the interactions of body temperature with sex and stimulus on escape speed. Bold values indicate statistically significant effects (*P* < 0.05).

Predictors		Estimate	Std. error	DF	*t*-value	*P*-value
	Intercept	0.409	0.017	135	24.63	**<0.001**
Fixed effects	Body Temperature (°C)	0.057	0.015	199	3.866	**<0.001**
	Stimulus Type (Strong vs. Weak)	−0.049	0.016	149	3.075	**0.003**
	Sex (Female vs. Male)	0.010	0.018	73	0.533	0.595
	Body Mass	0.020	0.010	79	2.056	**0.043**
	Orientation (head vs. tail)	−0.015	0.016	201	0.935	0.351
	Body Temperature × Stimulus Type	−0.026	0.016	171	1.642	0.102
	Body Temperature × Sex	0.004	0.016	184	0.247	0.805
	**Groups**	**Variance**	**SD**			
Random effect	Individual ID (*n* = 89)	0.001	0.027			
	Residual	0.013	0.113			

Marginal *R*^2^/Conditional *R*^2^ was 0.211/0.254.

DF values were calculated using the Satterthwaite approximation, which adjusts for the random effects structure and the correlations between observations.

### Does the temperature-dependence of antipredator behavior differ between the sexes in nymphs?

**Figure 3:**
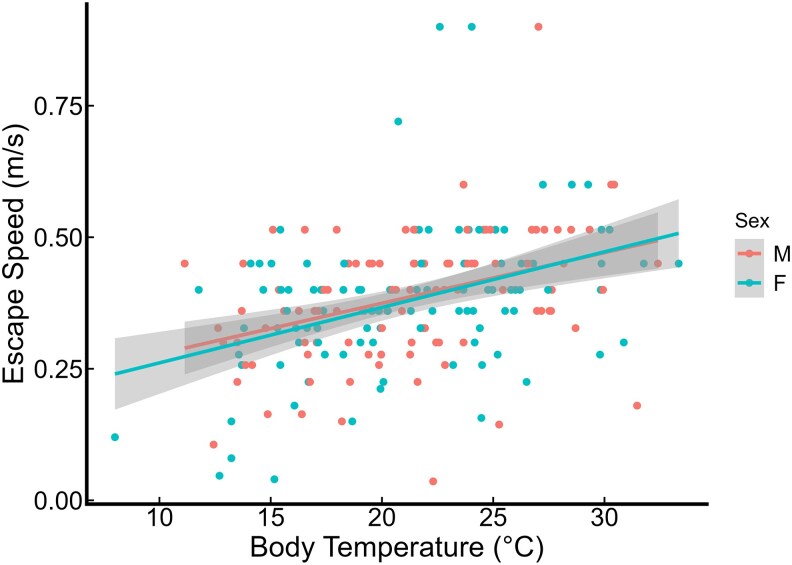
The relationship between body temperatures and escape speed in male and female nymphs. Red points and lines correspond to females, and blue points and lines to males. Shaded areas indicate 95% confidence intervals.

Unlike our previous study on adult crickets (Li et al. 2025), nymphs did not exhibit a similar interaction effect between sex and body temperature on escape speed (Table 1, Fig. 3).

## Discussion

Our experiments revealed that in their natural environment, crickets exhibit different escape speeds in response to varying intensities of predator cues. This suggests that rather than simply running as fast as they can whenever they perceive a threat, these insects are optimizing how they use their escape capabilities in relation to different risk levels. It is possible that faster running uses more energy, or that it creates a risk of injury, but further work to examine metabolic or other potential costs is needed to determine the nature of any speed/cost tradeoff.

Contrary to our initial expectations based on the threat-sensitivity hypothesis, the stronger vibrational stimulus was associated with slower running speeds relative to the weaker stimulus. One possible explanation is that field crickets may interpret strong vibrations as being generated by larger or slower-moving animals, which are less likely to represent immediate predation threats, rather than by small, agile predators. In this case, a very rapid escape response may not be necessary; entering the burrow within half a second may already provide sufficient safety, because the next footfall of a large mammal typically occurs after a longer interval.

Variation in the intensity of our threat cue across individuals may arise due to natural variation in soil composition. We minimized this potential source of noise by standardizing stimulus delivery: the ball was dropped from a fixed height of 10 cm behind the burrow entrance in all trials. Given that crickets typically position themselves within 4 cm of the burrow entrance, spatial differences in stimulus perception are likely negligible. This is supported by findings from Cocroft et al. (2006), who reported minimal effects of plant species, individuals, or distance on signal properties in a vibrational communication study on the treehopper (*Umbonia crassicornis*), with only ∼1% variation in signal length across distances (Cocroft et al. 2006). Although the cercal system (located at the posterior end of the cricket's abdomen) is highly sensitive to air currents and substrate vibrations (Shimozawa et al. 1998), our analysis showed that variation in the crickets’ orientation relative to the stimulus source did not significantly affect their escape speed. In a previous study, the effect of orientation on adult flee speed was stronger, but still also nonsignificant (Li et al. 2025).

We did not observe an interaction effect between sex and body temperature affecting escape speed in nymphs, which contrasts with the findings of our previous experiment on adults (Li et al. 2025). This discrepancy may reflect the fact that nymphs of both sexes are expected to focus on foraging and development, prioritizing their chances of survival. In contrast, male and female adults are likely to face different tradeoffs between fitness-related traits (Flatt and Heyland 2011). For example, male adults spend a significant amount of time outside their burrows singing to attract a mate (Rodríguez-Muñoz et al. 2025) and also frequently engage in fights with other males (Fisher et al. 2019), both of which are energetically demanding and expose them to risks of predation. In comparison, females produce and individually oviposit hundreds of eggs which may create a different balance of costs and benefits of escape behaviors.

Although crickets were randomly allocated to treatment groups in our experiment, we found that individuals receiving a weaker stimulus had lower body temperatures compared with the strong stimulus group, with the difference approaching statistical significance (see Table S1). Given that higher temperatures are associated with faster running speeds in insects (figure 2, 3) (Hurlbert et al. 2008), this imbalance should have biased the results in favor of faster running in the strong stimulus group. The fact that the crickets in the weak stimulus group were still significantly faster suggests that the magnitude of the difference we observed is a conservative estimate of the true effect of stimulus intensity on escape speed.

While body temperature and stimulus strength significantly influenced escape responses in our model, considerable variation remains unexplained (Marginal *R*^2^/Conditional *R*^2^ was 0.211/0.254). This likely reflects factors we did not measure. These include an individual's physiological state, such as activity level, hunger, metabolism (Lagos 2017). Micro-environmental variation may also play a role; soil texture and moisture can affect cue transmission and locomotion efficiency (Polajnar et al. 2015; Hill et al. 2019). Additionally, consistent behavioral traits like boldness or risk sensitivity could influence escape responses (Sih et al. 2004; Niemelä et al. 2012). Prior experience with predators or repeated exposure to stimuli might further alter responsiveness through habituation or sensitization (Adamo et al. 1995; Zuk et al. 2006). Future studies could explicitly investigate these factors to better understand individual variation in escape behavior.

### Predation risk allocation and energy optimization

Our findings support the predation risk allocation hypothesis, which predicts that antipredator responses should be context-dependent (Lima 1998; Lima and Bednekoff 1999). Animals can possess the ability to assess their risk of being preyed upon and incorporate this information into their antipredator decisions (Lima and Dill 1990). These decisions can be influenced by the intensity, frequency, and characteristics of predator signals (Lima and Dill 1990; Palmer and Packer 2021; Brown and Godin 2023). For instance, Lapiedra and his colleagues using lizards (*Anolis sagrei*) experimentally established populations on 8 small islands either with or without a major ground predator *Leiocephalus carinatus*, and found that lizards’ engaged in more exploratory behavior in the absence of predators, and avoided the ground in their presence (Lapiedra et al. 2018). Their study found that on predator islands, selection on behavior is stronger than selection on morphology, which suggests that large fitness costs and benefits are involved in modifying the level of response to predation cues.

The predation risk allocation hypothesis also intersects with hypotheses about prey energy optimization strategies under different predation risks (Lima and Bednekoff 1999). For example, a study on the snail (*Tegula funebralis*) found that they climb in response to chemical cues from crabs actively feeding on conspecific snails but not when exposed to cues from nonfeeding crabs (Jacobsen and Stabell 2004). Furthermore, a study on 2 species of damselfish (*Pomacentrus spp.*) illustrated that if an individual's energy budget is limited (eg, rising metabolic demands under rising ambient temperature), less resources are allocated to escape behavior (Warren et al. 2017).

Prey may also evolve heightened sensitivity to specific predator signals over time, as predicted by Helfman (1989) threat-sensitivity hypothesis (Helfman 1989; Jacobsen and Stabell 2004; Crawford et al. 2012). For instance, a study on greater siren (*Siren lacertina*) found that their avoidance behavior (reversing direction following cue detection) was higher in response to specialist predator and novel animal cues and lowest in response to generalist predator cues (Crawford et al. 2012).

### Optimal antipredator responses in ectotherms and climate change

Global temperatures are predicted to rise significantly over the next decades (IPCC 2023). There is clear potential for this to lead to higher average body temperatures in terrestrial ectotherms (Buckley et al. 2015; Gardner et al. 2024b), which may enhance antipredator performance. This may give insects an advantage over their homeothermic vertebrate predators whose body temperature is fixed. However, elevated temperatures might also compel ectotherms to allocate more energy to behavioral thermoregulation, or to limit periods of activity, thereby limiting resources available for investment in antipredator behaviors (Gilbert and Miles 2017; Warren et al. 2017; Gardner et al. 2024a). For instance, higher temperatures may force insects to reduce their frequency of rapid running to avoid heat stress (Gilbert and Miles 2017). This could place prey in a predicament under the dual pressures of predation and climate stress. The interaction between predator signal intensity and climate warming on insect antipredator behavior warrants further investigation. Future research should explore how long-term climate trends will shape predator-prey interactions.

## Supplementary Material

arag029_Supplementary_Data

## Data Availability

Analyses reported in this article can be reproduced using the data provided by (Li et al. 2026).
