# Supplementary material for: Wild crickets can adjust escaping speed under varying predation cues
Source: Behav Ecol. 2026 Mar 11;37(3):arag029. doi: 10.1093/beheco/arag029 (PMC13012820; doi:10.1093/beheco/arag029)
Supplement: arag029_Supplementary_Data [file arag029_supplementary_data.zip › Addtional Information of Wild Attack Event.docx]

**Additional Information of Wild Attack Event Video**

This video was recorded using an infra-red digital video camera in the meadow and edited to slightly reduce the playback speed, as the original recording was captured at 2 frames per second.

To better observe the crickets' escape process from predators, we recommend viewing the video at 0.8x speed or slower.
