# Supplementary material for: Wild crickets can adjust escaping speed under varying predation cues
Source: Behav Ecol. 2026 Mar 11;37(3):arag029. doi: 10.1093/beheco/arag029 (PMC13012820; doi:10.1093/beheco/arag029)
Supplement: arag029_Supplementary_Data [file arag029_supplementary_data.zip › Supplementary Material.docx]

**Relationships between body temperature and other traits**

We examined whether body temperature was associated with body mass (used here as a proxy for body size), orientation, and stimulus category. To do this, we fitted a separate mixed-effects model with these variables as predictors of body temperature. The results revealed a significant positive effect of body mass (see Table S1), indicating that larger individuals tended to have higher body temperatures. This suggests that variation in body size may contribute to individual differences in body temperature.

Table S1. Results of a linear mixed-effects model for the influence of predictors on body temperature.

| **Predictors** |  | **Estimate** | **Std. Error** | **DF** | **t-value** | **p-value** |
| --- | --- | --- | --- | --- | --- | --- |
|  | Intercept | 20.37 | 0.578 | 179 | 35.27 | **<0.001** |
| Fixed | Stimulus Type (Strong vs. Weak) | 1.014 | 0.568 | 128 | 1.785 | 0.077 |
|  | Body Mass | 1.754 | 0.402 | 77 | 4.366 | **<0.001** |
| Effects | Orientation (Posterior vs. Anterior) | 0.597 | 0.636 | 193 | 0.939 | 0.349 |
|  | **Groups** | **Variance** | **SD** |  |  |  |
| Random | Individual ID  (n=89) | 6.462 | 2.542 |  |  |  |
| effect | Residual | 16.25 | 4.031 |  |  |  |

Marginal R² / Conditional R² was 0.127/ 0.375

DF values were calculated using the Satterthwaite approximation, which adjusts for the random effects structure and the correlations between observations.
